# *Candida* Biofilms and the Host: Models and New Concepts for Eradication

**DOI:** 10.1155/2012/845352

**Published:** 2011-11-14

**Authors:** Hélène Tournu, Patrick Van Dijck

**Affiliations:** ^1^Laboratory of Molecular Cell Biology, Institute of Botany and Microbiology, Katholieke Universiteit Leuven, Flanders, 3001 Leuven-Heverlee, Belgium; ^2^Department of Molecular Microbiology, VIB, Kasteelpark Arenberg 31, Flanders, 3001, Leuven-Heverlee, Belgium

## Abstract

Biofilms define mono- or multispecies communities embedded in a self-produced protective matrix, which is strongly attached to surfaces. They often are considered a general threat not only in industry but also in medicine. They constitute a permanent source of contamination, and they can disturb the proper usage of the material onto which they develop. This paper relates to some of the most recent approaches that have been elaborated to eradicate *Candida* biofilms, based on the vast effort put in ever-improving models of biofilm formation *in vitro* and *in vivo*, including novel flow systems, high-throughput techniques and mucosal models. Mixed biofilms, sustaining antagonist or beneficial cooperation between species, and their interplay with the host immune system are also prevalent topics. Alternative strategies against biofilms include the lock therapy and immunotherapy approaches, and material coating and improvements. The host-biofilm interactions are also discussed, together with their potential applications in *Candida* biofilm elimination.

## 1. Introduction

Biofilms, adherent microbial communities embedded in a polymer matrix, are common in nature. However, they are also a persistent cause of hygiene problems in the food industry and in the medical field [1]. Biofilms result from a natural tendency of microbes to attach to biotic or abiotic surfaces, which can vary from mineral surfaces and mammalian tissues to synthetic polymers and indwelling medical devices, and to further grow on these substrates [2–4]. Candidiasis, caused most frequently by *Candida albicans*, and to a lesser extent by *C. glabrata*, *C. tropicalis,* or *C. parapsilosis*, is often associated with the formation of biofilms on the surface of medical devices and tissues [5]. *Candida albicans* is a dimorphic fungus and is part of the commensal human micoflora. It is also an opportunistic pathogen of the human body when its proliferation is not controlled by the host immune system. It is one of the most often identified agents in nosocomial infections and is capable of invading virtually any site of the human host, from deep tissues and organs, to superficial sites such as skin and nails, to medical implants and catheters [6]. *C. albicans* biofilm development has been characterized in various model systems both *in vitro* and *in vivo* [7–9] and consists of distinct phases. The initial step consists of the adhesion of fungal cells of the yeast form to the substrate. It is followed by a phase of cell filamentation and proliferation, which results in the formation of multiple layers of sessile cells of different morphologies, including pseudohyphal and hyphal cells. The next step of maturation results in a complex network of cells embedded in extracellular polymeric material, composed of carbohydrates, proteins, hexosamine, phosphorus and uronic acid, as well as host constituents in natural settings [10]. There is indeed evidence that host glycoproteins, nucleic acids, and cells, such as neutrophils, may participate in the maturity of the matrix, in particular on mucosal sites [11–13]. The establishment of the biofilm extracellular matrix (ECM) represents a unique characteristic of biofilms. Quantity and composition of the matrix vary from one species to another and in different sites of infection depending on environmental cues, such as nutrient availability and mechanical stimuli [14–17]. Matrix synthesis by *Candida* biofilm cells has been shown to be minimal in static conditions in comparison to dynamic environments [10], aggravating biofilm formation on mucosal and abiotic sites where there is a fluid flow, such as on the oral mucosa, the urethra, or central venous catheters. The last step, dispersion of cells from a biofilm, plays a key part in the biofilm developmental cycle as it is associated with candidemia and disseminated invasive disease [18]. 

Pathogenic microbes that build biofilms are potential causes of constant infections that defy the immune system and resist antimicrobial treatment, partly due to the matrix-inherent limited exposure of the cells within a biofilm to these types of immunological and medical arsenals [19–22]. Other mechanisms of biofilm resistance have been suggested, such as slow growth, differential regulation of the cell metabolic activity caused by nutrient limitation and stress conditions, and cell density [23–25]. In addition, the ability to adhere, as a unique prerequisite to form a biofilm, is a fast process, which makes the prevention of biofilm development difficult with the current antimicrobial tools and strategies. 

Biofilms are diverse communities and therefore vary depending on the microbe, the surface, and the colonization niche [5, 26–30]. This paper gives an update on the recent efforts made in establishing alternative means of eradication and also prevention of *Candida* spp. biofilms, by developing new models of biofilm formation in flow conditions, as well as high-throughput rapid screening analyses *in vitro*. Newly developed *in vivo* models anticipate a shift of interest towards mixed fungal-bacterial biofilms and their role in pathogenesis in mucosal infections in particular. Keeping in mind that there is no unique model representative of all biofilms, it remains quite a challenge to tackle biofilm inhibition. One of the most attractive perspectives is the development of antimicrobe materials, and the latest findings are presented here.

## 2. *Candida* Biofilm Models 

### 2.1. Models *In Vitro*

Biofilm formation is a multistep growth behaviour that results from complex physical, chemical, and biological processes [31, 32]. Because of the versatility of the milieu in which *Candida* biofilms can develop in the human host, from the oral cavity contributing to dental plaque formation to the blood stream in intravenous catheters and the urinary tract, it seemed necessary to reproduce *in vitro* as many conditions as possible to establish common and specific characteristics of *Candida* biofilm formation. In that respect, a multitude of *in vitro* studies has been described that relates to the impact of different types of substrate, nutritional supplies, in flow or static conditions, on adhesion and biofilm properties of several *Candida* species, and recent findings are presented next. An overview of the *in vitro* models available to study *Candida* biofilms is provided in Table 1.

#### 2.1.1. *Candida* Species and Substrates Specificities

While biofilm formation is a general characteristic of many microbes, biofilm features such as architecture, matrix composition, and resistance to antifungal drugs are species and substrate dependent. And examples that demonstrate variation in biofilm ability and structure are numerous. Some studies are discussed below, and in particular studies related to *Candida* biofilms formed on dental materials. Interest has indeed grown in investigating the role of *Candida* species and the effect of the type of material in the development of denture stomatitis [48]. For example, in a comparative study, cell counts analyses showed that saliva-coated discs harboured less *C. glabrata* cells than untreated discs, while the number of *C. albicans* cells was not affected by the saliva coating [33]. However, both species adhered better on hydroxyapatite (HA) surface than on two other types of dental material, polymethylmetacrylate and soft denture liner. Surprisingly, dual species experiments showed that *C. glabrata* displayed higher cell counts when grown in the presence of *C. albicans* than when grown alone. In contrast, hyphal development by *C. albicans* seemed to be reduced in the presence of *C. glabrata* in most of the conditions tested. These data may help understand the impact that *Candida* species may have on each other, as mixed species communities are being identified in clinical samples [49]. In another case study, using discs as support for biofilm formation *in vitro*, HA substrate appeared to be less prone to *Candida* adherence than acrylic denture, porcelain, or polystyrene when not coated with saliva [34]. In addition, the effect of serum and similar materials on biofilm development of *C. albicans* clinical isolates was also evaluated *in vitro* [35]. Disc coupons made of polycarbonate, polystyrene, stainless steel, polytetrafluoroethylene (also known as Teflon), polyvinyl chloride (PVC), or HA were used in a high throughput assay. For all surfaces tested, the presence of serum increased biofilm formation. However, in absence of serum, Teflon supported higher biofilm production than any other material, likely due to its high roughness and hydrophobicity properties. 

The differential ability to form biofilm of 84 strains from several *Candida* species, including *C. albicans*, *C. glabrata*, *C. krusei*, *C. tropicalis*, and *C. parapsilosis,* was assessed on clinical materials, such as Teflon and PVC. All species, with the exception of *Candida glabrata,* favoured Teflon [50]. In this study, *C. glabrata* together with *C. krusei* strains were not highly proficient in forming dense biofilms, as quantified by colony-forming units. Moreover, *C. parapsilosis* strains showed the least uniformity in the ability to form biofilm, followed by *C. tropicalis* and *C. albicans*. While some variability in the ability to form biofilms between strains of *C. albicans* has been documented *in vitro*, a study by MacCallum et al. [51] revealed that biofilm formation *in vitro* did not significantly vary between strains of the four major clades of *C. albicans*, classified according to single-nucleotide polymorphisms determinations and analysis of DNA repeat sequences [52]. However, high variation in the ability to form biofilm among strains of *C. parapsilosis* and less extensive biofilm formation by *C. glabrata* specimens has been illustrated in a few studies by crystal violet staining and confocal laser scanning microscopy [36–38]. Strain-dependent variation in biofilm formation was also observed among isolates of two genetically nonidentical classes of *C. parapsilosis*, namely, *C. orthopsilosis* and *C. metapsilosis* [39, 53]. All three species could form biofilms, but metabolic activity of biofilm cells differed between strains of the same species. However, conflicting data with different isolates reported the inability of *C. orthopsilosis* and *C. metapsilosis* to form biofilm in polystyrene 96-well plate assay *in vitro* [54, 55]. Biofilm formation among *C. parapsilosis sensu strictu* strains was also found to vary according to the geographical regions and the body sites from which the isolates came from [56]. Isolates from blood and cerebrospinal fluid seemed more prone to form biofilms than isolates from nails, catheters, and mucosa. Overall, these data suggest a high variability in biofilm ability of strains of *C. parapsilosis* and related species, perhaps due to inadequate models or to an intrinsic poor ability to establish the biofilm growth by these species. 

In a Calgary biofilm model adapted to *Candida* spp., *C. krusei* developed the largest biofilm mass in comparison to *C. albicans*, *C. glabrata*, *C. dubliensis,* and *C. tropicalis* [41]. This model, allowing 80 biofilms to be formed at once, seemed to be very favourable to *C. krusei* biofilm development as biofilms of that species constituted of thick multilayered structures composed of pseudohyphal cells, while the other species formed sparse biofilms. 

In a last example of novel *in vitro* models of biofilm formation on various soft contact lenses, analyses revealed differences in hyphal content and architecture of the fungal keratitis causative agents *Fusarium* and *C. albicans* [57]. Polymers such as balafilcon A and galyfilcon A were favourable to filamentous growth of *C. albicans*, while others such as etafilcon A and lotrafilcon A sustained biofilms formed mainly of yeast cells. In addition, differences in biofilm formation were also observed between peripheral and central regions of the lenses, with dense biofilms formed preferentially in the centres of the lenses. Although a direct relationship between the lens ionic charge and water content and the ability of fungi to form biofilm could not be established, these data confirm previous findings that irregular surface texture of materials affect both cellular morphology and biofilm mass [58].

#### 2.1.2. Synthetic Media and Flow Systems Mimicking *In Vivo* Conditions

The physiological specificity of infection sites is also an important factor, and efforts have been made to reproduce some major environmental cues *in vitro*, such as mimicking the blood flow or the urine. Biofilms grown in synthetic urine medium were comparable to those grown in the commonly used cell culture RPMI medium [59]. And time course studies revealed that the development of both types of biofilm followed a similar pattern, with an initial adherence phase, followed by growth, proliferation, and maturation. The biofilms differed slightly in their architecture, as biofilms grown in synthetic urine medium seemed to be less complex and less dense, with a larger proportion of yeast cells rather than elongated cells. Increased nutritional supply promoted biofilm formation in another model of artificial urine medium, highlighting once again the importance of reproducing as closely as possible the physiological conditions to gain relevant information [60]. *C. tropicalis* biofilms were also characterized in artificial urine medium, on urinary catheters in a flow model [61]. Cells were able to colonize the catheters in the presence of the artificial urine medium and to detach from these silicone catheters, illustrating their capacity to colonize distal sites. 

Biofilms grown in static conditions have been predominantly studied, in comparison to flow-based systems, due to a low cost, a rapid processing of large number of samples, and limited technical requirements. However, in order to maintain their niches in dynamic environments, biofilms *in vivo* endure shear forces generated by the constant flow of physiological fluids [62]. Gene expression analyses revealed only a marginal difference between biofilms grown in static conditions, such as microtiter plates or serum-treated catheters, and those grown in a flow system in microfermentors [44]. Interestingly, the biofilm transcriptomes were not strongly affected by factors such as nutrient flow and aerobiosis, in contrast to the gene expression of free-living cells. However, a few studies indicated that biofilms grown under flow conditions, in CDC reactors or modified Robbins devices, contain more extracellular matrix and more biomass [10, 43, 45]. Mature biofilms formed in a flow of replenishing nutrients consist of a dense network of yeast cells, pseudohyphae, and hyphal cells. In a simple flow model, using a silicone strip placed in a conical tube, *C. albicans* biofilms grew thicker than biofilms grown in static conditions, and grew faster as an 8-hour-grown flow biofilm had similar biomass as a 24-hour-grown static biofilm [46]. The authors speculated that uninterrupted food supply prohibited adverse conditions, such as nutrient starvation and toxic accumulation, and hence promoted rapid cell proliferation. A parallel study, using a rotating disc system (RDS) to impose shear forces at physiological levels to biofilms developed on catheter pieces, illustrated similar results as biofilms under shear stress grew thinner but denser than those in no-flow conditions [47]. In the RDS model, less cells adhered at first, but by 24 h biofilms displayed similar metabolic activity and dry weight as those obtained in the static model. Suggestions that explained the increased growth rate in shear conditions included an increased rate of maturation in these conditions and a natural selection of more robust cells capable of withstanding the fluid friction by growing faster. 

#### 2.1.3. High-Throughput Biofilm Models

Another important aspect of *in vitro* biofilm modelling is the development of high-throughput systems of particular interest in the large-scale screening of antibiofilm molecules. Most studies so far have made use of the 96-well microtiter plate assay [40]. In this model, biofilms are formed directly on the bottom of the wells, and the quantification method is based on the ability of sessile living cells to reduce tetrazolium salt (XTT) to water-soluble orange formazan compounds. In an effort to upscale biofilm production, a *C. albicans* biofilm chip system (*Ca*BChip) has recently been developed by Srinivasan et al. [42]. The high-density microarray platform is composed of more than 700 independent and uniform nanobiofilms encapsulated in a collagen matrix and provides the first miniature biofilm model for *C. albicans*. Despite the several-thousand-fold miniaturization, the biofilms formed on the chip displayed phenotypic characteristics, such as a multilayer of yeast, pseudohyphae and hyphal cells, and a high level of antifungal drug resistance, consistent with those of biofilms formed by standard methods. However, echinocandins were not proficient to eradicate biofilm in this system, potentially due to their binding to the collagen matrix. In a second generation of the biofilm chip, other nonprotein matrices will be investigated. While this system steps-up the number of biofilms that can be produced at once in static conditions, the next step may be to develop high-throughput flow biofilm systems adapted to *Candida* spp. Such a tool has been described based on a device comprised of microfluidic channels that provide fluid flow to 96 individual bacterial biofilms [63]. The effects of antimicrobial agents on the biofilms were rapidly screened, and viability was quantified by fluorescence measurements. These high-throughput techniques will certainly contribute greatly to the discovery of novel antibiofilm molecules.

### 2.2. *In Vivo* Models of *Candida* Biofilms

#### 2.2.1. Biofilm Models on Inert Substrates

**Figure 1:**
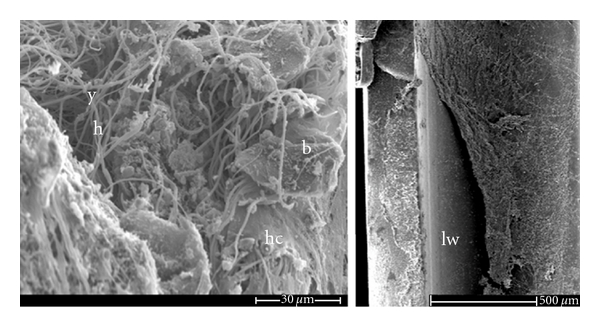
Scanning electron microscopy images of wild type *Candida albicans* biofilms developed *in vivo* in the denture model (left panel) and in the subcutaneous model (right panel). Elements such as hyphal cells (h), yeast cells (y), bacterial cells (b), host cells (hc) and catheter lumen wall (lw) are highlighted. Images were adapted from the work of Nett et al. [67], and S. Kucharíková and P. Van Dijck (MCB Laboratory, VIB, K.U. Leuven, unpublished data), respectively.

*In vivo* models are undisputedly required to appreciate the hostile environment that conditions biofilm formation (Table 2). A few *Candida* biofilm models, mostly associated to catheter infections, have been developed in several rodents, giving insights on the *in vivo* biofilm structure and the efficacy of various antifungal agents [70]. The catheter-related *in vivo* biofilm models resulted in biofilm formation within 24 h and consisted of complex structures of yeast and elongated cells embedded in extracellular matrix, similar to those observed in *in vitro* model systems [8]. While susceptibility to azoles was reduced in these models, liposomal amphotericin B lock therapy and treatment with caspofungin or chitosan proved to be efficient against *in vivo* biofilms [64, 65, 71]. Central venous catheter models (CVCs) are also useful for the investigation of the kinetics and occurrence of dissemination of the microorganisms to other organs, demonstrated by colonisation by *C. albicans* of the kidneys in the rat model [8]. In addition, the development of a CVC model in mice will allow comparison to other modes of infection, in particular to the commonly used disseminated candidiasis by tail vein infection. A murine model for catheter-associated candiduria was recently developed and illustrated the role of *Candida* biofilms in the persistence of the urinary tract infection [66]. It also outlined differences between murine and human catheter-related candiduria in terms of bladder inflammation and fungal burden in the urine. In another catheter-related *Candida*-associated infection model, we developed a subcutaneous foreign body system suitable for *C. albicans* [9]. This model, of nondisseminated nature, allowed the study of biofilm development for long periods of time (Figure 1) but required the use of immunosuppression treatment of the animals due to the high inflammatory response associated with implant of foreign devices. However, efficacy of the echinocandin anidulafungin, by intraperitoneal injections, was demonstrated against *C. albicans* biofilm in this *in vivo* system [72]. These *in vivo *models are all suited for further study of novel antifungal therapies and for the use of novel material technologies, including less adherent surfaces and material coating with fixed or releasing antifungal agents (see the next section).

A relatively cost- and time-effective *Candida* biofilm model on acrylic denture material, which does not require the *ex vivo* mold process, was illustrated recently [67]. In this rat model, biofilms developed between the hard palate and the denture material, following *Candida* inoculation in that 1 mm space (Figure 1). Fungal invasion of the palate and the tongue and neutrophils infiltration also occurred, indicating that the model was consistent with that of acute human denture stomatitis. Interestingly, the denture model offers the possibility to study mixed biofilm structure and behaviour in response to antimicrobial treatments, as the biofilms were composed of both bacterial and fungal cells. Finally, biofilms developed on the denture model were inherently resistant to fluconazole, in accordance with previous findings [8, 72], but also to the echinocandin micafungin, in contrast with previous investigations performed in a different model [73]. A plausible explanation suggested by the authors is that the mixed biofilm nature combined with the specific site of infection, the oral cavity, is the cause of that antifungal resistance. An alternative rat model of *Candida*-associated denture stomatitis recently described differs by the use of animal-fitted devices [68]. In this system, a removable part of the device makes the replacement of the infected device a relatively easy step. These models promise to deliver an alternative mean of testing novel antibiofilm molecules. 

#### 2.2.2. Biofilm Models on Biotic Surfaces

Tools and models to study biofilm formation developed on implanted materials are numerous and indicative of the increased medicinal use of such implants. Biofilms formed on live surfaces are much less characterized, yet they are recognized as causing or aggravating numerous chronic diseases [74]. Besides dental plaques, few reports have investigated biofilm development in clinical samples. Biotic biofilms are poorly understood as tissue samples are sparse and not easily accessible. The oral cavity is an accessible *in vivo* model for studying protein-surface interactions and has been well characterized for bacterial biofilm [75]. A mucosal model of oropharyngeal candidiasis was recently proposed to characterise *C. albicans* mucosal biofilms *in situ* in mice [12]. Keratin, originating from desquamating epithelial cells, constituted a large proportion of the biofilm matrix. First evidence was given that epithelial cells, neutrophils, and commensal oral bacteria co-exist within the fungal mucosal biofilm developed on mouse tongue. Bacteria were mostly found on the apical part of the biofilm, and very few were seen to invade the tongue epithelium layer. This model highlights the complexity of mucosal biofilms, as host elements and commensal organisms contribute in an active or passive manner to the structure of the biofilms. 

*C. albicans* can also form biofilms on the vaginal mucosa, illustrated by two *in vivo* and *ex vivo* models in immunocompetent estradiol-treated mice [69]. *C. albicans* vaginal biofilms consisted of yeast and hyphal cells embedded in extracellular material, illustrated by ConA staining of the interspersed matrix. In the *ex vivo* model using vaginal explants, no exogenous nutrients were provided, yet biofilms were formed most likely by scavenging host nutrients. 

Host-pathogen interactions in biofilm settings have not yet been elucidated, but comparison between these models promises to identify model-specific fungal and host elements.

### 2.3. Mixed Species *Candida* Biofilms

The relative contribution and the role of bacteria-*Candida* interactions in the pathogenesis of mucosal infections are yet to be established. However, there is clear evidence that multimicrobial interactions have a central role in the context of human disease [76]. For example, microbial diversity was illustrated in a biofilm-related infection of the urinary tract [77]. Out of 535 clinical samples of urinary catheters, *Candida* spp. were identified among the 39 different microbial taxa isolated. Single-species samples represented 12.5% only. *C. albicans* was isolated in 141 samples, and other *Candida* species were present in other 82 samples. Biofilm formation ability of each isolated strain was quantified *in vitro*, yet not in an artificial urine medium, and cut-off values were used to define no, weak, intermediate, and strong biofilm producers. *C. tropicalis* isolates were the strongest biofilm producers among the *Candida* species. Certain species of bacteria did not show biofilm formation ability in this study. These data illustrates the fact that, in multispecies biofilms, some have a great potential to cause biofilm-based infections, while others may be more passive members of the structured community. Commensalism, mutual cooperation, and antagonism make the interactions within mixed biofilms complex [78, 79]. A summary of bacteria-*Candida* interactions and their effect on fungal development is provided in Table 3. Bacteria can interact with *C. albicans* cells within mixed biofilms, and in particular with hyphal cells. The methicillin-resistant Gram-positive *Staphylococcus aureus* had the highest hyphal association, in comparison to *S. epidermidis*, *Strepococcus pyogenes*, *Pseudomonas aeruginosa*, *Bacillus subtilis,* and *Escherichia coli* in decreasing order, respectively [80]. However, interaction between *S. aureus* and *C. albicans* did not result in reduced or altered biofilm viability. In another study, addition of bacteria to preformed *Candida* biofilms *in vitro* had an antagonistic effect on biofilm cell mass, often in a cell-density-dependent manner [81]. With all inoculums tested, *P. aeruginosa* reduced significantly the fungal biofilm mass when added during the first few hours of biofilm development. In a different experimental assay, preformed bacterial biofilms significantly reduced adhesion and biofilm growth of *C. albicans* [82]. Moreover, simultaneous addition of bacteria and *C. albicans* cells showed that in all cases fungal adhesion was decreased, whereas bacterial biomasses were not affected. 

Hypotheses of synergistic relationships between microbes have been suggested, and in particular within mixed biofilm communities [83]. For example, bacterial adhesion was observed on the tongue mucosa of *C. albicans*-infected animals but not of noninfected animals, in a mucosal model of oropharyngeal candidiasis [12]. Synergistic cooperation can also perturb susceptibility to antimicrobial treatment. For example, *S. aureus* resistance to vancomycin was enhanced in mixed biofilms with viable *C. albicans* cells, whereas susceptibility of the fungal cells to the antifungal amphotericin B was not altered [84]. Binding of the fungus to the bacterial cells occurs via the *Candida*-specific adhesin proteins, including Als3, Eap1, and Hwp1, as demonstrated by heterologous expression of these cell wall proteins in the model yeast *Saccharomyces cerevisiae* [85]. The role of adhesins in single- and multispecies biofilm formation is not discussed here but can be found in previous reports [86–88].

## 3. Antibiofilm Strategies: Research and Development

The current therapies against fungal diseases [96], employing one of the five classes of antifungals (polyenes, pyrimidine analogues, allylamines, azoles, and echinocandins) administrated orally or intravenously, are not discussed in this paper. Each antifungal compound has advantages and limitations related to its spectrum of activity and mode of action. The susceptibility of *Candida* biofilms to the current therapeutic agents remains low, with the exception of the echinocandins [97, 98]. However, these compounds have been employed in different approaches, such as lock therapy or material coating as releasing agent. These alternative methods and their perspective of usage are discussed below. 

### 3.1. Lock Therapy Approach and Prevention against Catheter-Related Blood Stream Infections

Nosocomial infections associated with medical devices represent a large proportion of all cases of hospital-acquired infections [99]. In particular, insertion of any vascular catheter can result in a catheter-related infection, as microorganisms can colonise catheter external and internal surfaces. Some of the favourite niches of colonisation of *Candida* spp. include indeed vascular and urinary catheters and ventricular assist devices, which can be accompanied with high mortality rates [100]. Adherence to the catheter surface, facilitated by host proteins such as fibronectin and fibrinogen, can then lead to biofilm formation [101]. The antimycotic lock therapy approach is currently recommended and employed in treating catheter-related bloodstream infections (CRBSI), in particular for long-term catheters, according to the Infectious Diseases Society of America guidelines [102]. However, lock therapeutic treatment is pathogen– specific as catheter removal is recommended for CRBSI caused by *Candida *species and *Staphylococcus aureus*. The lock therapy involves the instillation of high doses of an antimicrobial agent (from 100- to 1000-fold the minimal inhibitory concentration, (MIC)) directly into the catheter in order to “lock” it for a certain period of time (from hours to days) [103].

Few reports are currently available on the usage of antifungal lock solutions in clinical practice, but they seem to indicate the curative effect of this kind of treatment [104, 105]. *In vitro* studies are more prevalent at the moment and seem to also favour the use of antifungal lock therapy to eliminate *Candida* spp. biofilms, and in particular with the usage of echinocandins [106]. For example, biofilm metabolic activity formed on silicone by *C. albicans* and *C. glabrata* could be effectively reduced by a 12 h lock treatment with micafungin (at 100–500x MIC), which was shown to persist for up to 3 days [107]. Caspofungin had an intermediate effectiveness in the same study, as its activity did not persist as long against *C. glabrata* biofilms. While these results are promising for potential use of the lock technique to treat infected catheters, 100% biofilm inhibition could not be achieved. Sterilization of catheters was obtained *in vivo* by lock treatment with amphotericin B lipid complex (ABLC) in a rabbit model of catheter-associated *C. albicans* biofilm [108]. However, in this study, the lock solution was administrated a few hours a day for a prolonged period of time (7 days). Synergistic antibiofilm combinations, used as lock solutions, between classical antimicrobial agents and other compounds such as the mucolytic agent N-acetylcysteine, ethanol, or the chelating agent EDTA, are also effective against *S. epidermidis* and *C. albicans* individual and mixed biofilms [109]. In a similar approach, recent results suggest that the combination of antibacterial agents with Gram-positive activity, including doxycycline and tigecycline, with known antifungals, such as AMB, caspofungin, and fluconazole, can be useful for the treatment of *C. albicans* biofilms [110, 111].

The prevention of CRBSI has also been the focus of research and randomized controlled trials [112]. In a systematic assessment, Hockenhull et al. [113] showed the clinical effectiveness of CVCs treated with anti-infective agents (AI-CVC) in preventing CRBSI. While trials are still required to determine the most cost and clinical-effective anti-infective product, the routine usage of AI-CVC will often be limited if appropriate use of other practical care behaviour is not employed in intensive care units. Antifungal impregnated CVCs have been tested in animal models. The echinocandin caspofungin was employed to prevent *C. albicans* biofilm formation in a biofilm model in mice. *C. albicans* biofilm formation was greatly reduced in CVCs that had been pretreated for 24 h with high doses of caspofungin. The dissemination to the kidneys was also reduced by such therapy [65]. Similarly, the use of chitosan, a polymer isolated from crustacean exoskeletons, as a pretreatment of catheters to prevent *C. albicans* biofilm formation was validated in a CVC biofilm *in vivo* model [71]. The use of lock technique or preventive impregnation of antifungals in combating catheter-associated infection seems promising, but not yet convincing on a cost effective point of view as huge doses are still needed to eradicate fungal growth.

### 3.2. Material Coating and Novel Antibiofilm Surfaces

A developing field of research focuses on the usage of modified materials or coated surfaces to prevent adherence and biofilm development. Implant materials are prone to biofilm formation affecting health in general and duration of the implant in particular. Surface characteristics, such as surface roughness, surface free energy, and chemistry, can influence the type and the feature of the biofilms [114, 115]. For example, *C. albicans* adhesion is enhanced if the roughness of the denture materials is increased [116]. It is nowadays conceivable that coatings may be engineered to promote selective adhesion, with possible attachment to cell tissue (for implant in bone contact) but not to microbes. They may also address the second phase of biofilm development involving quorum sensing, by inhibiting cell-cell communication signals [117, 118]. Biomaterial modifications as a way to prevent biofilm development have been the focus of intense research, in particular in the field of bacterial biofilms [119], but the latest findings on their impact on *Candida* biofilms are discussed next. 

#### 3.2.1. Surface Modifications

Surface properties of medical devices constitute a major factor contributing not only to the stability in the body but also to their performance and lifetime *in vivo* and their colonization by microorganisms. In that matter, albumin adhesion is beneficial since it has been shown to prevent binding of microorganisms, while fibrinogen has the opposite effect [120]. Chemical grafting of polyethylene and polypropylene surfaces, functionalized with cyclodextrins, yielded a change in protein adsorption profile of these polymers, by promoting adsorption of albumin and reducing adhesion of fibrinogen to the material surface [121]. In addition, these modified substrates incorporated well the antifungal agent miconazole, leading to reduced biofilm formation by *C. albicans in vitro*. Modified polyethylene and silicone rubbers proved to be very efficient in inhibiting *C. albicans* biofilm formation *in vitro* [122]. These cytocompatible materials were also capable of releasing for several hours considerable amount of an anionic antimicrobial drug, nalidixic acid, suggesting their use as drug-eluting systems. 

Modifications of polyurethanes dental biomaterials by addition of surface-modifying end groups were successfully employed to manage *C. albicans* biofilm formation [123]. In addition, correlation between contact angle and biofilm formation was surface dependent. Increased hydrophobicity resulted in increased metabolic activity of the biofilms grown on polyetherurethane, while they inversely correlated for biofilms formed on polycarbonate surfaces. Addition of 6% polyethylene oxide to Elastane 80A showed to be the best combination as no biofilm could be observed on that surface. Biofilms on voice prostheses consist of mixed populations that can include *C. albicans*. Modification of the silicone surface of the prostheses has been employed to limit *C. albicans* colonization, as opposed to incorporation of antimicrobial agents in order to avoid the occurrence of resistance [124]. Silicone disks grafted with C1 and C8 alkyl side chains reduced adherence and biofilm formation of *C. albicans* by up to 92%. Longer side chains did not show as good results, and combinations of quaternizing agents did not work synergistically either. Similarly, grafting of cationic peptides, such as the salivary peptide Hst5 and synthetic variants, onto silicone rubber, inhibited biofilm formation by up to 93%, in a peptide-dependent manner [125]. 

#### 3.2.2. Surface Coatings

Fungicidal or fungistatic materials have been employed to fabricate or coat the surfaces of medical devices and have a great potential in reducing or eliminating the incidence of biofilm-related infections. Dental resin material coated with thin-film polymer formulations containing the polyene antifungals nystatin, amphotericin B, or the antiseptic agent chlorhexidine, were used in *C. albicans* biofilm assays [126]. Biofilm reduction was the greatest on chlorhexidine containing polymers, while the other formulations were much less efficient. Similarly, multilayered polyelectrolyte thin films containing an antifungal *β*-peptide incorporated within the layers of the films inhibited the growth (and hyphal formation) of *C. albicans* by 74% after 2 h of contact [127]. 

The polysaccharide dextran is widely used in medicine and is also one of the main components of dental plaque. Cross-linked dextran disks soaked with amphotericin B solutions, described as amphogel, kills fungi within 2 hours of contact and can be reused for almost 2 months without losing its efficacy against *C. albicans* [128]. This antifungal material is biocompatible and could be used to coat medical devices to prevent microbial attachment. It was recently used for local antifungal therapy in the form of injectable cross-linking hydrogels [129]. Nitric oxide can antagonise cell proliferation by signalling rather than by toxic effect. It regulates bacterial biofilm dispersal and has also been employed in releasing xerogel to attenuate *C. albicans* adherence and biofilm formation [130]. The nitric-oxide-based method is still at the experimental level, due to poor water solubility and stability. 

Coating of medical material surfaces has been employed and tested with several types of coating molecules, including the naturally occurring polymer chitosan and antimicrobial peptides such as Histatin 5 (Hst5). Surfaces coated with the polymer reduced the viable cell number in biofilms by more than 95%, in the case of *C. albicans* and also for many bacteria such as *Staphylococcus aureus* [131]. Chitosan, which is proficient against a wide range of pathogenic microbes, disrupts cell membranes as cells settle on the surface. The use of such polymer offers a biocompatible tool for further coating design of medical devices. Acrylic disks precoated with Hst5 prove to be efficient in inhibiting biofilm formation of *C. albicans*, especially in the later stage of development, while biofilm sensitivity to the antimicrobial peptide was the same as the one of free-living cells [132]. The utility and potential of selected peptides, as therapeutic molecules, including the *β*-glucan synthesis inhibitors, the histidine-rich peptides, and the LL-37 cathelicidin family are being determined and could be used as coating compounds against adherence and biofilm formation [133, 134]. 

The possible applications of biomaterial modification remain to be clearly established and approved. Shift from a commensal bacterial biofilm to a more pathogenic biofilm involving *Candida* spp. in the oral cavity for instance is believed to be more influenced by mucosal inflammation and the general well-being of the host than on the nature and surface properties of the material itself [135]. However, development of materials that can fully abolish microbial adherence is a promising perspective against biofilm formation. The discrepancy between antimicrobial coatings killing the biofilm-proficient organisms and antimicrobial releasing coatings to prevent biofilm formation is a current issue.

### 3.3. Quorum Sensing Molecules and Natural Byproducts

Adhesion and biofilm formation by *C. albicans* cells can be modulated by physical and chemical signals from the oral bacterium *Streptococcus gordonii *[91]. Indeed, most *Streptococcus* species possess the antigen I/II, a cell-wall-anchored protein receptor that mediates binding to *C. albicans*. Moreover, *C. albicans* hyphal and biofilm development are greatly enhanced by *S. gordonii*, which also relieved the fungal cells from the repressing effect of the quorum sensing molecule farnesol [91]. Farnesol, a sesquiterpene and signalling molecule produced by *C. albicans*, represses biofilm formation *in vitro* [136]. Conversely, tyrosol, a 2-(4-hydroxyphenyl) ethanol derivative of tyrosine, accelerates hypha production in the early stages of biofilm development and is secreted at least 50% more by biofilm cells than by planktonic cells [137]. Several studies demonstrated that farnesol actually increases fungal pathogenicity in animal models, potentially by interfering with normal progression of cytokine induction [138–140]. Analogs of farnesol have been identified that fail to induce pathogenicity and yet retain farnesol ability to block hyphal development [141]. While these analogs did not protect mice from candidiasis, they may be of interest in biofilm inhibition. Indeed, a number of molecules with farnesol-like activity, that can induce the shift to the yeast form of growth, have been identified in Gram-negative bacteria. For instance, the signalling molecule, homoserine lactone, produced by *Pseudomonas aeruginosa* represses *C. albicans* filamentation [142]. *P. aeruginosa *also produces several phenazines that exhibit antifungal activity against *C. albicans* [143]. Uptake of the phenazines generated reactive oxygen species production and led to fungal cell death. In mixed biofilms, binding of the toxins to the fungal cells has a negative influence on *C. albicans* growth.

In a different approach, Valle et al. [144] demonstrated that the use of nonantibiotic molecules, such as polysaccharides, produced by competitive commensal organisms can antagonize biofilm formation. A better knowledge of the microbial community behaviour and in particular of the interaction between commensal and pathogen organisms would help to combat predominance of the infectious or disease causative agents. In this scheme, natural products produced by cells within a biofilm contribute to the dynamic of the community and may play an antiadhesion role for nonwanted other microorganisms [145]. Bacterial lipopolysaccharides also modulate adhesion and biofilm ability of several *Candida* species, in an interspecies-dependent manner [146]. It is not known how mixed populations affect the host immune response in response to infection. The overall population behaviour results from a potential selective advantage to either or both species. While communication is the key, interpretation is the code. Identification and alterations of the communication signals would certainly result in a better understanding of how species coexist and permit a better control of biofilm formation [147]. Targeting quorum sensing molecules or associated signalling mechanisms is an open field of research at present, but the use of quorum quenching enzymes or quorum sensing inhibitors naturally produced by other species could help in the finding of novel antibiofilm agents [148, 149].

### 3.4. Host Responses to Biofilms: Perspective of Immunotherapy

With the number of people considered at high risk for microbial infections constantly increasing, immunotherapy seems to offer a great potential despite the complexity of the interaction between the host defence system and the pathogen [150]. The ability of human pathogens, such as *Candida* spp., to cause infections depends on a constant and sometimes discontinuous battle between the pathogen and the host immune system [151]. Recognition of *Candida*-specific pathogen-associated molecular patterns (PAMPs) by dedicated pattern recognition receptors (PRRs) such as Toll-like receptors and lectins activates the innate effector cells (macrophages, dendritic cells, and neutrophils), which in turn produce a variety of soluble factors, including cytokines and chemokines [152]. However, little is yet known about the interactions between human phagocytes and *Candida* spp. biofilms, while immunotherapeutic treatment against candidiasis has been undertaken [153, 154]. Chandra et al. [155] demonstrated that adherent peripheral blood mononuclear cells (PBMCs) enhanced the ability of *C. albicans* to form biofilm. They also observed that phagocytosis of the fungal cells within a biofilm did not occur while their free-living counterparts were phagocytosed. These data defined the novel concept that *Candida* biofilms seem to have an immunosuppressive effect. Inactivated PBMCs on the other hand did not induce this enhanced growth behaviour, nor did lipopolysaccharide-activated PBMCs, suggesting that the stimulated biofilm formation resulted from (a) *Candida*-biofilm-induced secretory factor(s). Indeed, the cytokine profile of PBMCs following coculture with planktonic or biofilm cells of *C. albicans* differed greatly, with IL-1*β* as the cytokine most highly overexpressed by contact with biofilms. Supporting these data, a recent study showed that phagocytes alone induced much less damage to biofilms than they did to free-living cells or to resuspended biofilm cells, which lacked the overall structure of biofilms and most of the matrix [156]. Using confocal laser scanning microscopy, Katragkou and coworkers deducted that human phagocytes looked like unstimulated cells, presenting a rounded shape when in presence of biofilms. This was also confirmed by a reduced cytokine production in a biofilm-phagocyte coculture, compared to a planktonic cells-phagocytes mix. Phagocytes appeared entrapped within the structured network of cells and matrix and were unable to internalize cells within biofilms. Moreover, *C. albicans* and *C. parapsilosis* biofilms were more susceptible to the additive effects between phagocytic host defence and the echinocandin anidulafungin than to each separately and to the combination of the azole voriconazole with phagocytes [156, 157]. These data validate the findings that echinocandins can influence host cell interactions with biofilm [158]. 

Pathogens have evolved many mechanisms of defence to avoid being recognized by the host environment [159–161]. *C. albicans* can evade immune attack by masking its cell wall *β*-glucan component, a potent pro-inflammatory signature carbohydrate, under a thick layer of mannoproteins. Clear evidence showed that exposing the *β*-glucans by treatment with the antifungal drug caspofungin elicited a stronger immune response [158]. These data suggest that echinocandin treatment may enhance immunity [162]. Masking of *β*-glucans depends on a complex network of cell wall remodelling, and targeting these regulatory processes may identify novel antifungal possibilities. For example, disruption of the MAPK pathway regulated by the extracellular signal-induced Cek1 kinase triggered a greater *β*-glucan exposure, which resulted in an enhanced immune response compared to the wild-type strain [163]. There are conflicting data regarding the role of the *β*-glucan receptor Dectin-1, expressed widely on phagocytes, in antifungal immunity [164]. However, studies suggested that Dectin-1 is required for fungal killing and induction of early inflammatory responses. These findings are of interest for biofilm recognition by the immune system, as *β*-1,3-glucans are found in high amounts in the extracellular matrix of *Candida* biofilms *in vitro* and *in vivo* [10, 12, 165]. Biofilms developed on soft tissue are associated with infiltration of the infected sites by neutrophils, which can then confer innate immune protection [166]. In *C. albicans*, Hyr1, encoding a GPI-anchored cell wall protein, has been shown to confer resistance to neutrophil killing *in vitro* and in the oral mucosal tissue biofilm model [12, 167]. In addition, vaccination with a recombinant Hyr1p protected mice against hematogenously disseminated candidiasis. Immunotherapeutic strategies, such as vaccination, anti-*Candida *antibodies, and cytokine therapy, are under investigation to treat *Candida* infections [168]. However, their applicability in treating biofilm-related infections is still in a preliminary state. In that framework, recent data showed that pretreatment of *C. albicans* cells with antibodies targeting the complement receptor 3-related protein led to reduced adhesion and biofilm formation *in vitro* [169]. In another study, anti-*C. albicans* antibodies from chicken egg yolk were employed as antiadherent molecules [170]. While the adherence of *C. albicans* was reduced, biofilm inhibition was only observed in absence of serum, as the activity of the antibody was very much reduced against germ tubes, of which the formation is induced in the presence of serum. *In vivo* studies of the antibody-based approach remain to be investigated in the context of biofilms.

## 4. Concluding Remarks

The large panel of biofilm models suitable for *Candida* research highlights the diversity of niches in which the fungus can develop ranging from biotic to abiotic surfaces. However, the role and nature of host-pathogen interactions during biofilm formation are only starting to get unveiled. The search for an antibiofilm treatment is a complex subject which requires improved knowledge of the pathogen itself, and also of the host response to adhesion and biofilm formation, the properties of the substrates onto which biofilm develop, and the interactions within microbial communities. The field of chemoinformatics may assist the development of novel antibiofilm compounds, based on already identified good candidate molecules [171]. This approach may also reveal better coating agents for material surfaces that would persist long periods of time *in vivo*. The use of natural compounds, from dietary plants or probiotics, may also be considered as they are better tolerated by humans.

## Figures and Tables

**Table 1:** Examples of *Candida* biofilm models *in vitro*.

Models *in vitro *	Device	Used for
Closed systems (discontinuous growth conditions over time (nutrient depletion, accumulation of secondary metabolites))	(i) 96-well polystyrene microtiter plate (ii) Discs/pieces of catheter in 6- to 24-well plate (discs made of silicone, polyurethane, polycarbonate, polystyrene, stainless steel, Teflon, polyvinyl chloride, hydroxyapatite, and porcelain)	Easy and widespread use: comparative analyses between strains and species [33–39] to antifungal susceptibility tests [40]
	(iii) Calgary biofilm device (80 pegs immersed into a standard 96-well plate)	Biofilm formation studies by different *Candida* species [41]
	(iv) *Candida* biofilm chip (several hundreds nanobiofilms encapsulated in collagen and formed on a glass slide treated to obtained a monolayer of hydrophobic coating)	High-throughput biofilm studies [42]

Flow systems (Continuous growth conditions)	(i) CDC biofilm reactor (24 biofilms can be formed simultaneously)	Comparative analysis of biofilm quantification methods [43]
	(ii) Microfermentors (biofilms formed on a Thermanox slide glued to a glass spatula)	Gene expression analyses [44]
	(iii) Modified Robbins device (adapted to hold several individual discs)	Study of the effects of shear forces and nutrient supplies on *C. albicans* biofilm formation [45]
	(iv) Flow biofilm model (silicone elastomer strip placed into a polypropylene conical tube)	Study of *C. albicans* biofilm development, architecture, and drug resistance [46]

Shear stress conditions	Rotating disc system (silicone catheter devices placed under a shear force of 350 revolutions per minute)	*C. albicans* biofilm architecture and development [47]

**Table 2:** *Candida* biofilms *in vivo* models.

Models *in vivo *	Device	Developed in
Catheter-associated models	(i) Central venous system	Rat [8], rabbit [64], mouse [65]
	(ii) Candiduria model	Mouse [66]
	(iii) Subcutaneous foreign body system (biofilms developed after 2 to 6 days in infected implated catheter fragments)	Rat (immunosuppressed before and during biofilm development) [9]

*Candida*-associated denture stomatitis models	(i) Acrylic denture material attached to the hard palate (biofilms developed between the hard palate and the device)	Rat (immunosuppressed on day of infection) [67]
	(ii) Custom fitted denture system (cast fabrication of a fixed part that is attached to the posterior palate and a removable part fitted to the anterior palate)	Rat [68]

Mucosal model of oropharyngeal candidiasis	Biofilms developed on the tongue after infection by swabbing and drinking water contaminated with *Candida* cells	Mouse (immunosuppressed on day of infection) [12]

Vaginitis model	*In vivo* and *ex vivo* models	Mouse (treated with estradiol prior infection) [69]

**Table 3:** Interspecies relationship with *Candida* spp. growth and biofilm development.

Bacterial species	Effect on *C. albicans* hyphal growth	Effect on *Candida* biofilm
*Staphylococcus aureus* (+)	Associates to hyphal cells (56%) [80]	No antagonistic effect in dual biofilms with *C. albicans *(BacLight LIVE/DEAD assay) [80]

*Staphylococcus epidermidis* (+)	Associates to hyphal cells (25%) [80]	Reduced adhesion and biofilm formation by a glycocalyx producer strain (CFU counts) [82]

*Streptococcus pyogenes* (+)	Associates to hyphal cells (25%) [80]	

*Streptococcus mutans and Streptococcus intermedius*	*S. mutans* inhibits hyphal formation [89, 90]	No significant effect on biofilm viability at densities ranging from 6.25·10^5^ to 1·10^7^ cells/mL (bacteria added to preformed 3-hour-old biofilms; polystyrene *in vitro* model; CFUs analyses) [81]

*Streptococcus gordonii* (+)	Stimulates hyphal growth [91]	Promotes mixed biofilms with *C. albicans* [91]

*Pseudomonas aeruginosa* (−)	(i) Associates to hyphal cells (17%) [80] (ii) Reduced hyphal growth in *C. albicans*-*P. aeruginosa* dual biofilms [81] (iii) Binds hyphae and kill *C. albicans* [92]	(i) Reduced adhesion and biofilm formation by a nonglycocalyx producer strain (CFU counts) [82] (ii) Reduction of biofilm mass ranging from 40% to 80% in a density-dependent manner [90] (iii) Mutual biofilm inhibition between *Pa* and *C. albicans*, *C. krusei* and *C. glabrata*; decreased biofilm formation of *C. parapsilosis* and *C. tropicalis* in presence of *Pa*; increased CFUs of *Pa* in presence of *C. tropicalis* [93]

*Escherichia coli* (−)	Associates to hyphal cells (5.7%) [80]	(i) Reduction of biofilm mass ranging from 50% to 80% [81] (ii) Mutual decrease in biofilm cell mass between *Ec* and *C. albicans*; inhibition of biofilm development by *C. tropicalis*, *C. parapsilosis*, *C. krusei,* and *C. dubliniensis*; increased *Ec* cell numbers within *C. tropicalis* and *C. dubliniensis* biofilms [94]

*Lactobacillus acidophilus*		Inhibition of viable biofilm cell mass by 40% [81]

*Bacillus subtilis*	Associates to hyphal cells (2.5%) [80]	

*Actinomyces israelii* (+)		Some inhibition of biofilm at high densities [81]

*Prevotella nigrescens and Porphyromonas gingivalis*	Inhibition of *C. albicans* hyphal development [95]	Reduction of *C. albicans* biofilms, only at high densities [81]

*Klebsiella pneumoniae, Serratia marcescens, and Enterobacter cloacae*		Decreased biofilm formation (CFU counts) [82]

## References

[1] Meyer B (2003). Approaches to prevention, removal and killing of biofilms. *International Biodeterioration and Biodegradation*.

[2] Dongari-Bagtzoglou A (2008). Pathogenesis of mucosal biofilm infections: challenges and progress. *Expert Review of Anti-Infective Therapy*.

[3] Donlan RM (2002). Biofilms: microbial life on surfaces. *Emerging Infectious Diseases*.

[4] Davis LE, Cook G, William Costerton J (2002). Biofilm on ventriculoperitoneal shunt tubing as a cause of treatment failure in coccidioidal meningitis. *Emerging Infectious Diseases*.

[5] Ramage G, Martínez JP, López-Ribot JL (2006). *Candida* biofilms on implanted biomaterials: a clinically significant problem. *FEMS Yeast Research*.

[6] Martinez LR, Fries BC (2010). Fungal biofilms: relevance in the setting of human disease. *Current Fungal Infection Reports*.

[7] Ramage G, VandeWalle K, Wickes BL, López-Ribot JL (2001). Characteristics of biofilm formation by *Candida albicans*. *Revista Iberoamericana de Micologia*.

[8] Andes D, Nett J, Oschel P, Albrecht R, Marchillo K, Pitula A (2004). Development and characterization of an *in vivo* central venous catheter *Candida albicans* biofilm model. *Infection and Immunity*.

[9] Řičicová M, Kucharíková S, Tournu H (2010). *Candida albicans* biofilm formation in a new *in vivo* rat model. *Microbiology*.

[10] Al-Fattani MA, Douglas LJ (2006). Biofilm matrix of *Candida albicans* and *Candida tropicalis*: chemical composition and role in drug resistance. *Journal of Medical Microbiology*.

[11] Walker TS, Tomlin KL, Worthen GS (2005). Enhanced *Pseudomonas aeruginosa* biofilm development mediated by human neutrophils. *Infection and Immunity*.

[12] Dongari-Bagtzoglou A, Kashleva H, Dwivedi P, Diaz P, Vasilakos J (2009). Characterization of mucosal *Candida albicans* biofilms. *PLoS ONE*.

[13] Martins M, Uppuluri P, Thomas DP (2010). Presence of extracellular DNA in the *Candida albicans* biofilm matrix and its contribution to biofilms. *Mycopathologia*.

[14] d’Enfert C (2006). Biofilms and their role in the resistance of pathogenic *Candida* to antifungal agents. *Current Drug Targets*.

[15] Martinez LR, Casadevall A (2007). *Cryptococcus neoformans* biofilm formation depends on surface support and carbon source and reduces fungal cell susceptibility to heat, cold, and UV light. *Applied and Environmental Microbiology*.

[16] Mowat E, Williams C, Jones B, McChlery S, Ramage G (2009). The characteristics of *Aspergillus fumigatus* mycetoma development: is this a biofilm?. *Medical Mycology*.

[17] Singh R, Shivaprakash MR, Chakrabarti A (2011). Biofilm formation by zygomycetes: quantification, structure and matrix composition. *Microbiology*.

[18] Uppuluri P, Chaturvedi AK, Srinivasan A (2010). Dispersion as an important step in the *Candida albicans* biofilm developmental cycle. *PLoS Pathogens*.

[19] Kuchma SL, O’Toole GA (2000). Surface-induced and biofilm-induced changes in gene expression. *Current Opinion in Biotechnology*.

[20] Whiteley M, Bangera MG, Bumgarner RE (2001). Gene expression in *Pseudomonas aeruginosa* biofilms. *Nature*.

[21] Thomas DP, Bachmann SP, Lopez-Ribot JL (2006). Proteomics for the analysis of the *Candida albicans* biofilm lifestyle. *Proteomics*.

[22] Vediyappan G, Rossignol T, D’Enfert C (2010). Interaction of *Candida albicans* biofilms with antifungals: transcriptional response and binding of antifungals to beta-glucans. *Antimicrobial Agents and Chemotherapy*.

[23] Jayampath Seneviratne C, Wang Y, Jin L, Abiko Y, Samaranayake LP (2010). Proteomics of drug resistance in *Candida glabrata* biofilms. *Proteomics*.

[24] Nett JE, Sanchez H, Cain MT, Ross KM, Andes DR Interface of *Candida albicans* biofilm matrix-associated drug resistance and cell wall integrity regulation.

[25] Perumal P, Mekala S, Chaffin WL (2007). Role for cell density in antifungal drug resistance in *Candida albicans* biofilms. *Antimicrobial Agents and Chemotherapy*.

[26] Romero D, Kolter R (2011). Will biofilm disassembly agents make it to market?. *Trends in Microbiology*.

[27] Kumamoto CA (2002). Candida biofilms. *Current Opinion in Microbiology*.

[28] Blankenship JR, Mitchell AP (2006). How to build a biofilm: a fungal perspective. *Current Opinion in Microbiology*.

[29] Estrela AB, Heck MG, Abraham WR (2009). Novel approaches to control biofilm infections. *Current Medicinal Chemistry*.

[30] Espinel-Ingroff A (2009). Novel antifungal agents, targets or therapeutic strategies for the treatment of invasive fungal diseases: a review of the literature (2005–2009). *Revista Iberoamericana de Micologia*.

[31] Nett J, Andes D (2006). *Candida albicans* biofilm development, modeling a host-pathogen interaction. *Current Opinion in Microbiology*.

[32] Cos P, Toté K, Horemans T, Maes L (2010). Biofilms: an extra hurdle for effective antimicrobial therapy. *Current Pharmaceutical Design*.

[33] Pereira-Cenci T, Deng DM, Kraneveld EA (2008). The effect of *Streptococcus* mutans and *Candida glabrata* on *Candida albicans* biofilms formed on different surfaces. *Archives of Oral Biology*.

[34] Li L, Finnegan MB, Özkan S (2010). *In vitro* study of biofilm formation and effectiveness of antimicrobial treatment on various dental material surfaces. *Molecular Oral Microbiology*.

[35] Frade JP, Arthington-Skaggs BA (2011). Effect of serum and surface characteristics on *Candida albicans* biofilm formation. *Mycoses*.

[36] Silva S, Henriques M, Martins A, Oliveira R, Williams D, Azeredo J (2009). Biofilms of non-*Candida albicansCandida* species: quantification, structure and matrix composition. *Medical Mycology*.

[37] Kuhn DM, Chandra J, Mukherjee PK, Ghannoum MA (2002). Comparison of biofilms formed by *Candida albicans* and **Candida parapsilosis** on bioprosthetic surfaces. *Infection and Immunity*.

[38] Silva S, Negri M, Henriques M, Oliveira R, Williams DW, Azeredo J (2011). Adherence and biofilm formation of non-*Candida albicans Candida* species. *Trends in Microbiology*.

[39] Lattif AA, Mukherjee PK, Chandra J (2010). Characterization of biofilms formed by **Candida parapsilosis**, *C. metapsilosis*, and *C. orthopsilosis*. *International Journal of Medical Microbiology*.

[40] Pierce CG, Uppuluri P, Tristan AR (2008). A simple and reproducible 96-well plate-based method for the formation of fungal biofilms and its application to antifungal susceptibility testing. *Nature Protocols*.

[41] Parahitiyawa NB, Samaranayake YH, Samaranayake LP (2006). Interspecies variation in *Candida* biofilm formation studied using the Calgary biofilm device. *Acta Pathologica, Microbiologica et Immunologica Scandinavica*.

[42] Srinivasan A, Uppuluri P, Lopez-Ribot J, Ramasubramanian AK (2011). Development of a high-throughput *Candida albicans* biofilm chip. *PLoS ONE*.

[43] Honraet K, Goetghebeur E, Nelis HJ (2005). Comparison of three assays for the quantification of *Candida* biomass in suspension and CDC reactor grown biofilms. *Journal of Microbiological Methods*.

[44] García-Sánchez S, Aubert S, Iraqui I, Janbon G, Ghigo JM, D’Enfert C (2004). *Candida albicans* biofilms: a developmental state associated with specific and stable gene expression patterns. *Eukaryotic Cell*.

[45] Ramage G, Wickes BL, López-Ribot JL (2008). A seed and feed model for the formation of *Candida albicans* biofilms under flow conditions using an improved modified Robbins device. *Revista Iberoamericana de Micologia*.

[46] Uppuluri P, Chaturvedi AK, Lopez-Ribot JL (2009). Design of a simple model of *Candida albicans* biofilms formed under conditions of flow: development, architecture, and drug resistance. *Mycopathologia*.

[47] Mukherjee PK, Chand DV, Chandra J, Anderson JM, Ghannoum MA (2009). Shear stress modulates the thickness and architecture of *Candida albicans* biofilms in a phase-dependent manner. *Mycoses*.

[48] Bergendal T, Isacsson G (1983). A combined clinical, mycological and histological study of denture stomatitis. *Acta Odontologica Scandinavica*.

[49] Agwu E, Ihongbe JC, McManus BA, Moran GP, Coleman DC, Sullivan DJ Distribution of yeast species associated with oral lesions in HIV-infected patients in Southwest Uganda.

[50] Estivill D, Arias A, Torres-Lana A, Carrillo-Muñoz AJ, Arévalo MP (2011). Biofilm formation by five species of *Candida* on three clinical materials. *Journal of Microbiological Methods*.

[51] MacCallum DM, Castillo L, Nather K (2009). Property differences among the four major *Candida albicans* strain clades. *Eukaryotic Cell*.

[52] Odds FC (2010). Molecular phylogenetics and epidemiology of *Candida albicans*. *Future Microbiology*.

[53] Tavanti A, Davidson AD, Gow NAR, Maiden MCJ, Odds FC (2005). *Candida orthopsilosis* and *Candida metapsilosis* spp. nov. to replace **Candida parapsilosis** groups II and III. *Journal of Clinical Microbiology*.

[54] Song JW, Shin JH, Shin DH (2005). Differences in biofilm production by three genotypes of **Candida parapsilosis** from clinical sources. *Medical Mycology*.

[55] Tavanti A, Hensgens LAM, Ghelardi E, Campa M, Senesi S (2007). Genotyping of *Candida orthopsilosis* clinical isolates by amplification fragment length polymorphism reveals genetic diversity among independent isolates and strain maintenance within patients. *Journal of Clinical Microbiology*.

[56] Tavanti A, Hensgens LA, Mogavero S, Majoros L, Senesi S, Campa M (2010). Genotypic and phenotypic properties of **Candida parapsilosis* sensu strictu* strains isolated from different geographic regions and body sites. *BMC Microbiology*.

[57] Imamura Y, Chandra J, Mukherjee PK (2008). *Fusarium* and *Candida albicans* biofilms on soft contact lenses: model development, influence of lens type, and susceptibility to lens care solutions. *Antimicrobial Agents and Chemotherapy*.

[58] Chandra J, Kuhn DM, Mukherjee PK, Hoyer LL, McCormick T, Ghannoum MA (2001). Biofilm formation by the fungal pathogen *Candida albicans*: development, architecture, and drug resistance. *Journal of Bacteriology*.

[59] Uppuluri P, Dinakaran H, Thomas DP, Chaturvedi AK, Lopez-Ribot JL (2009). Characteristics of *Candida albicans* biofilms grown in a synthetic urine medium. *Journal of Clinical Microbiology*.

[60] Jain N, Kohli R, Cook E, Gialanella P, Chang T, Fries BC (2007). Biofilm formation by and antifungal susceptibility of *Candida* isolates from urine. *Applied and Environmental Microbiology*.

[61] Negri M, Silva S, Henriques M, Azeredo J, Svidzinski T, Oliveira R (2011). Candida tropicalis biofilms: artificial urine, urinary catheters and flow model. *Medical Mycology*.

[62] Busscher HJ, van der Mei HC (2006). Microbial adhesion in flow displacement systems. *Clinical Microbiology Reviews*.

[63] Benoit MR, Conant CG, Ionescu-Zanetti C, Schwartz M, Matin A (2010). New device for high-throughput viability screening of flow biofilms. *Applied and Environmental Microbiology*.

[64] Schinabeck MK, Long LA, Hossain MA (2004). Rabbit model of *Candida albicans* biofilm infection: lliposomal amphotericin B antifungal lock therapy. *Antimicrobial Agents and Chemotherapy*.

[65] Lazzell AL, Chaturvedi AK, Pierce CG, Prasad D, Uppuluri P, Lopez-Ribot JL (2009). Treatment and prevention of *Candida albicans* biofilms with caspofungin in a novel central venous catheter murine model of candidiasis. *Journal of Antimicrobial Chemotherapy*.

[66] Wang X, Fries BC (2011). A murine model for catheter-associated Candiduria. *Journal of Medical Microbiology*.

[67] Nett JE, Marchillo K, Spiegel CA, Andes DR (2010). Development and validation of an *in vivo Candida albicans* biofilm denture model. *Infection and Immunity*.

[68] Lee H, Yu A, Johnson CC, Lilly EA, Noverr MC, Fidel PL (2011). Fabrication of a multi-applicable removable intraoral denture system for rodent research. *Journal of Oral Rehabilitation*.

[69] Harriott MM, Lilly EA, Rodriguez TE, Fidel PL, Noverr MC (2010). *Candida albicans* forms biofilms on the vaginal mucosa. *Microbiology*.

[70] Coenye T, Nelis HJ (2010). *In vitro* and *in vivo* model systems to study microbial biofilm formation. *Journal of Microbiological Methods*.

[71] Martinez LR, Mihu MR, Tar M (2010). Demonstration of antibiofilm and antifungal efficacy of chitosan against candidal biofilms, using an *in vivo* central venous catheter model. *Journal of Infectious Diseases*.

[72] Kucharíková S, Tournu H, Holtappels M, Van Dijck P, Lagrou K (2010). In vivo efficacy of anidulafungin against mature *Candida albicans* biofilms in a novel rat model of catheter-associated candidiasis. *Antimicrobial Agents and Chemotherapy*.

[73] Kuhn DM, George T, Chandra J, Mukherjee PK, Ghannoum MA (2002). Antifungal susceptibility of *Candida* biofilms: unique efficacy of amphotericin B lipid formulations and echinocandins. *Antimicrobial Agents and Chemotherapy*.

[74] Hall-Stoodley L, Stoodley P (2009). Evolving concepts in biofilm infections. *Cellular Microbiology*.

[75] Hannig C, Hannig M (2009). The oral cavity—a key system to understand substratum-dependent bioadhesion on solid surfaces in man. *Clinical Oral Investigations*.

[76] Morales DK, Hogan DA (2010). *Candida albicans* interactions with bacteria in the context of human health and disease. *PLoS pathogens*.

[77] Holá V, Ruzicka F, Horka M (2010). Microbial diversity in biofilm infections of the urinary tract with the use of sonication techniques. *FEMS Immunology and Medical Microbiology*.

[78] Thein ZM, Seneviratne CJ, Samaranayake YH, Samaranayake LP (2009). Community lifestyle of *Candida* in mixed biofilms: a mini review. *Mycoses*.

[79] Shirtliff ME, Peters BM, Jabra-Rizk MA (2009). Cross-kingdom interactions: *Candida albicans* and bacteria. *FEMS Microbiology Letters*.

[80] Peters BM, Jabra-Rizk MA, Scheper MA, Leid JG, Costerton JW, Shirtliff ME (2010). Microbial interactions and differential protein expression in **Staphylococcus aureus* -Candida albicans* dual-species biofilms. *FEMS Immunology and Medical Microbiology*.

[81] Thein ZM, Samaranayake YH, Samaranayake LP (2006). Effect of oral bacteria on growth and survival of *Candida albicans* biofilms. *Archives of Oral Biology*.

[82] El-Azizi MA, Starks SE, Khardori N (2004). Interactions of *Candida albicans* with other *Candida* spp. and bacteria in the biofilms. *Journal of Applied Microbiology*.

[83] Ten Cate JM, Klis FM, Pereira-Cenci T, Crielaard W, De Groot PWJ (2009). Molecular and cellular mechanisms that lead to *Candida* biofilm formation. *Journal of Dental Research*.

[84] Harriott MM, Noverr MC (2009). *Candida albicans* and *Staphylococcus aureus* form polymicrobial biofilms: effects on antimicrobial resistance. *Antimicrobial Agents and Chemotherapy*.

[85] Nobbs AH, Margaret Vickerman M, Jenkinson HF (2010). Heterologous expression of *Candida albicans* cell wall-associated adhesins in *Saccharomyces cerevisiae* reveals differential specificities in adherence and biofilm formation and in binding oral *Streptococcus gordonii*. *Eukaryotic Cell*.

[86] Klotz SA, Gaur NK, De Armond R (2007). *Candida albicans* Als proteins mediate aggregation with bacteria and yeasts. *Medical Mycology*.

[87] Silverman RJ, Nobbs AH, Vickerman MM, Barbour ME, Jenkinson HF (2010). Interaction of *Candida albicans* cell wall Als3 Protein with *Streptococcus gordonii* SspB adhesin promotes development of mixed-species communities. *Infection and Immunity*.

[88] Liu Y, Filler SG (2011). Candida albicans Als3, a multifunctional adhesin and invasin. *Eukaryotic Cell*.

[89] Jarosz LM, Deng DM, van der Mei HC, Crielaard W, Krom BP (2009). *Streptococcus* mutans competence-stimulating peptide inhibits *Candida albicans* hypha formation. *Eukaryotic Cell*.

[90] Vílchez R, Lemme A, Ballhausen B (2010). *Streptococcus mutans* inhibits *Candida albicans* hyphal formation by the fatty acid signaling molecule trans-2-decenoic acid (SDSF). *ChemBioChem*.

[91] Bamford CV, D’Mello A, Nobbs AH, Dutton LC, Vickerman MM, Jenkinson HF (2009). *Streptococcus gordonii* modulates *Candida albicans* biofilm formation through intergeneric communication. *Infection and Immunity*.

[92] Hogan DA, Kolter R (2002). *Pseudomonas-Candida* interactions: an ecological role for virulence factors. *Science*.

[93] Bandara HMHN, Yau JYY, Watt RM, Jin LJ, Samaranayake LP (2010). *Pseudomonas aeruginosa* inhibits *in-vitro Candida* biofilm development. *BMC Microbiology*.

[94] Bandara HMHN, Yau JYY, Watt RM, Jin LJ, Samaranayake LP (2009). *Escherichia coli* and its lipopolysaccharide modulate *in vitro Candida* biofilm formation. *Journal of Medical Microbiology*.

[95] Nair RG, Anil S, Samaranayake LP (2001). The effect of oral bacteria on *Candida albicans* germ-tube formation. *Acta Pathologica, Microbiologica et Immunologica Scandinavica*.

[96] Denning DW, Hope WW (2010). Therapy for fungal diseases: opportunities and priorities. *Trends in Microbiology*.

[97] Jabra-Rizk MA, Falkler WA, Meiller TF (2004). Fungal biofilms and drug resistance. *Emerging Infectious Diseases*.

[98] Lewis K (2008). Multidrug tolerance of biofilms and persister cells. *Current Topics in Microbiology and Immunology*.

[99] Kojic EM, Darouiche RO (2004). *Candida* infections of medical devices. *Clinical Microbiology Reviews*.

[100] Cauda R (2009). Candidaemia in patients with an inserted medical device. *Drugs*.

[101] Christner M, Franke GC, Schommer NN (2010). The giant extracellular matrix-binding protein of *Staphylococcus epidermidis* mediates biofilm accumulation and attachment to fibronectin. *Molecular Microbiology*.

[102] Mermel LA, Allon M, Bouza E (2009). Clinical practice guidelines for the diagnosis and management of intravascular catheter-related infection: 2009 update by the infectious diseases society of America. *Clinical Infectious Diseases*.

[103] Carratalà J (2002). The antibiotic-lock technique for therapy of “highly needed” infected catheters. *Clinical Microbiology and Infection*.

[104] Viale P, Petrosillo N, Signorini L, Puoti M, Carosi G (2001). Should lock therapy always be avoided for central venous catheter-associated fungal bloodstream infections?. *Clinical Infectious Diseases*.

[105] Angel-Moreno A, Boronat M, Bolaños M, Carrillo A, González S, Pérez Arellano JL (2005). Candida glabrata fungemia cured by antibiotic-lock therapy: case report and short review. *Journal of Infection*.

[106] Donlan RM (2008). Biofilms on central venous catheters: is eradication possible?. *Current Topics in Microbiology and Immunology*.

[107] Cateau E, Berjeaud JM, Imbert C (2011). Possible role of azole and echinocandin lock solutions in the control of *Candida* biofilms associated with silicone. *International Journal of Antimicrobial Agents*.

[108] Mukherjee PK, Long L, Kim HG, Ghannoum MA (2009). Amphotericin B lipid complex is efficacious in the treatment of *Candida albicans* biofilms using a model of catheter-associated *Candida* biofilms. *International Journal of Antimicrobial Agents*.

[109] Venkatesh M, Rong L, Raad I, Versalovic J (2009). Novel synergistic antibiofilm combinations for salvage of infected catheters. *Journal of Medical Microbiology*.

[110] Miceli MH, Bernardo SM, Lee SA (2009). In vitro analyses of the combination of high-dose doxycycline and antifungal agents against *Candida albicans* biofilms. *International Journal of Antimicrobial Agents*.

[111] Ku TSN, Palanisamy SKA, Lee SA (2010). Susceptibility of *Candida albicans* biofilms to azithromycin, tigecycline and vancomycin and the interaction between tigecycline and antifungals. *International Journal of Antimicrobial Agents*.

[112] Edgeworth J (2009). Intravascular catheter infections. *Journal of Hospital Infection*.

[113] Hockenhull JC, Dwan KM, Smith GW (2009). The clinical effectiveness of central venous catheters treated with anti-infective agents in preventing catheter-related bloodstream infections: a systematic review. *Critical Care Medicine*.

[114] Quirynen M, Bollen CM (1995). The influence of surface roughness and surface-free energy on supra- and subgingival plaque formation in man. A review of the literature. *Journal of Clinical Periodontology*.

[115] Teughels W, Van Assche N, Sliepen I, Quirynen M (2006). Effect of material characteristics and/or surface topography on biofilm development. *Clinical Oral Implants Research*.

[116] Radford DR, Sweet SP, Challacombe SJ, Walter JD (1998). Adherence of *Candida albicans* to denture-base materials with different surface finishes. *Journal of Dentistry*.

[117] Xiong Y, Liu Y (2010). Biological control of microbial attachment: a promising alternative for mitigating membrane biofouling. *Applied Microbiology and Biotechnology*.

[118] Bruellhoff K, Fiedler J, Möller M, Groll J, Brenner RE (2010). Surface coating strategies to prevent biofilm formation on implant surfaces. *International Journal of Artificial Organs*.

[119] Busscher HJ, Rinastiti M, Siswomihardjo W, van der Mei HC (2010). Biofilm formation on dental restorative and implant materials. *Journal of Dental Research*.

[120] Anderson JM, Rodriguez A, Chang DT (2008). Foreign body reaction to biomaterials. *Seminars in Immunology*.

[121] Nava-Ortiz CAB, Burillo G, Concheiro A (2010). Cyclodextrin-functionalized biomaterials loaded with miconazole prevent *Candida albicans* biofilm formation in vitro. *Acta Biomaterialia*.

[122] Contreras-García A, Bucioa E, Brackmanc G, Coenyec T, Concheirob A, Alvarez-Lorenzob C (2011). Biofilm inhibition and drug-eluting properties of novel DMAEMA-modified polyethylene and silicone rubber surfaces. *Biofouling*.

[123] Chandra J, Patel JD, Li J (2005). Modification of surface properties of biomaterials influences the ability of *Candida albicans* to form biofilms. *Applied and Environmental Microbiology*.

[124] de Prijck K, de Smet N, Coenye T, Schacht E, Nelis HJ (2010). Prevention of *Candida albicans* biofilm formation by covalently bound dimethylaminoethylmethacrylate and polyethylenimine. *Mycopathologia*.

[125] De Prijck K, De Smet N, Rymarczyk-Machal M (2010). *Candida albicans* biofilm formation on peptide functionalized polydimethylsiloxane. *Biofouling*.

[126] Redding S, Bhatt B, Rawls HR, Siegel G, Scott K, Lopez-Ribot J (2009). Inhibition of *Candida albicans* biofilm formation on denture material. *Oral Surgery, Oral Medicine, Oral Pathology, Oral Radiology and Endodontology*.

[127] Karlsson AJ, Flessner RM, Gellman SH, Lynn DM, Palecek SP (2010). Polyelectrolyte multilayers fabricated from antifungal *β*-peptides: design of surfaces that exhibit antifungal activity against *Candida albicans*. *Biomacromolecules*.

[128] Zumbuehl A, Ferreira L, Kuhn D (2007). Antifungal hydrogels. *Proceedings of the National Academy of Sciences of the United States of America*.

[129] Hudson SP, Langer R, Fink GR, Kohane DS (2010). Injectable in situ cross-linking hydrogels for local antifungal therapy. *Biomaterials*.

[130] Privett BJ, Nutz ST, Schoenfisch MH (2010). Efficacy of surface-generated nitric oxide against *Candida albicans* adhesion and biofilm formation. *Biofouling*.

[131] Carlson RP, Taffs R, Davison WM, Stewart PS (2008). Anti-biofilm properties of chitosan-coated surfaces. *Journal of Biomaterials Science, Polymer Edition*.

[132] Pusateri CR, Monaco EA, Edgerton M (2009). Sensitivity of *Candida albicans* biofilm cells grown on denture acrylic to antifungal proteins and chlorhexidine. *Archives of Oral Biology*.

[133] Matejuk A, Leng Q, Begum MD (2010). Peptide-based antifungal therapies against emerging infections. *Drugs of the Future*.

[134] Tsai P-W, Yang C-Y, Chang H-T, Lan C-Y (2011). Human antimicrobial peptide LL-37 inhibits adhesion of *Candida albicans* by interacting with yeast cell-wall carbohydrates. *PLoS ONE*.

[135] Avon SL, Goulet JP, Deslauriers N (2007). Removable acrylic resin disk as a sampling system for the study of denture biofilms *in vivo*. *Journal of Prosthetic Dentistry*.

[136] Ramage G, Saville SP, Wickes BL, López-Ribot JL (2002). Inhibition of *Candida albicans* biofilm formation by farnesol, a quorum-sensing molecule. *Applied and Environmental Microbiology*.

[137] Alem MAS, Oteef MDY, Flowers TH, Douglas LJ (2006). Production of tyrosol by *Candida albicans* biofilms and its role in quorum sensing and biofilm development. *Eukaryotic Cell*.

[138] Navarathna DHMLP, Hornby JM, Hoerrmann N, Parkhurst AM, Duhamel GE, Nickerson KW (2005). Enhanced pathogenicity of *Candida albicans* pre-treated with subinhibitory concentrations of fluconazole in a mouse model of disseminated candidiasis. *Journal of Antimicrobial Chemotherapy*.

[139] Navarathna DHMLP, Hornby JM, Krishnan N, Parkhurst A, Duhamel GE, Nickerson KW (2007). Effect of farnesol on a mouse model of systemic candidiasis, determined by use of a DPP3 knockout mutant of *Candida albicans*. *Infection and Immunity*.

[140] Navarathna DHMLP, Nickerson KW, Duhamel GE, Jerrels TR, Petro TM (2007). Exogenous farnesol interferes with the normal progression of cytokine expression during candidiasis in a mouse model. *Infection and Immunity*.

[141] Shchepin R, Navarathna DHMLP, Dumitru R, Lippold S, Nickerson KW, Dussault PH (2008). Influence of heterocyclic and oxime-containing farnesol analogs on quorum sensing and pathogenicity in *Candida albicans*. *Bioorganic and Medicinal Chemistry*.

[142] Hogan DA, Vik A, Kolter R (2004). A *Pseudomonas aeruginosa* quorum-sensing molecule influences *Candida albicans* morphology. *Molecular Microbiology*.

[143] Morales DK, Jacobs NJ, Rajamani S, Krishnamurthy M, Cubillos-Ruiz JR, Hogan DA (2010). Antifungal mechanisms by which a novel *Pseudomonas aeruginosa* phenazine toxin kills *Candida albicans* in biofilms. *Molecular Microbiology*.

[144] Valle J, Da Re S, Henry M (2006). Broad-spectrum biofilm inhibition by a secreted bacterial polysaccharide. *Proceedings of the National Academy of Sciences of the United States of America*.

[145] Rendueles O, Travier L, Latour-Lambert P (2011). Screening of *Escherichia coli* species biodiversity reveals new biofilm- associated antiadhesion polysaccharides. *mBio*.

[146] Bandara HMHN, Lam OLT, Watt RM, Jin LJ, Samaranayake LP (2010). Bacterial lipopolysaccharides variably modulate in vitro biofilm formation of Candida species. *Journal of Medical Microbiology*.

[147] Njoroge J, Sperandio V (2009). Jamming bacterial communication: new approaches for the treatment of infectious diseases. *EMBO Molecular Medicine*.

[148] Macedo AJ, Abraham WR (2009). Can infectious biofilm be controlled by blocking bacterial communication?. *Medicinal Chemistry*.

[149] Lazar V Quorum sensing in biofilms—how to destroy the bacterial citadels or their cohesion/power?.

[150] Van Der Meer JWM, Van De Veerdonk FL, Joosten LAB, Kullberg B-J, Netea MG (2010). Severe *Candida* spp. infections: new insights into natural immunity. *International Journal of Antimicrobial Agents*.

[151] Bourgeois C, Majer O, Frohner IE, Tierney L, Kuchler K (2010). Fungal attacks on mammalian hosts: pathogen elimination requires sensing and tasting. *Current Opinion in Microbiology*.

[152] Seider K, Heyken A, Lüttich A, Miramón P, Hube B (2010). Interaction of pathogenic yeasts with phagocytes: survival, persistence and escape. *Current Opinion in Microbiology*.

[153] Kullberg BJ, Oude Lashof AML, Netea MG (2004). Design of efficacy trials of cytokines in combination with antifungal drugs. *Clinical Infectious Diseases*.

[154] Wozniak KL, Palmer G, Kutner R, Fidel PL (2005). Immunotherapeutic approaches to enhance protective immunity against *Candida* vaginitis. *Medical Mycology*.

[155] Chandra J, McCormick TS, Imamura Y, Mukherjee PK, Ghannoum MA (2007). Interaction of *Candida albicans* with adherent human peripheral blood mononuclear cells increases *C. albicans* biofilm formation and results in differential expression of pro- and anti-inflammatory cytokines. *Infection and Immunity*.

[156] Katragkou A, Kruhlak MJ, Simitsopoulou M (2010). Interactions between human phagocytes and *Candida albicans* biofilms alone and in combination with antifungal agents. *Journal of Infectious Diseases*.

[157] Katragkou A, Chatzimoschou A, Simitsopoulou M, Georgiadou E, Roilides E (2011). Additive antifungal activity of anidulafungin and human neutrophils against *Candida parapsilosis* biofilms. *Journal of Antimicrobial Chemotherapy*.

[158] Wheeler RT, Fink GR (2006). A drug-sensitive genetic network masks fungi from the immune system. *PLoS Pathogens*.

[159] Meiller TF, Hube B, Schild L (2009). A novel immune evasion strategy of *Candida albicans*: proteolytic cleavage of a salivary antimicrobial peptide. *PLoS ONE*.

[160] Luo S, Poltermann S, Kunert A, Rupp S, Zipfel PF (2009). Immune evasion of the human pathogenic yeast *Candida albicans*: pra1 is a Factor H, FHL-1 and plasminogen binding surface protein. *Molecular Immunology*.

[161] Gropp K, Schild L, Schindler S, Hube B, Zipfel PF, Skerka C (2009). The yeast *Candida albicans* evades human complement attack by secretion of aspartic proteases. *Molecular Immunology*.

[162] Perlin DS (2011). Current perspectives on echinocandin class drugs. *Future Microbiology*.

[163] Galán-Díez M, Arana DM, Serrano-Gómez D (2010). Candida albicans *β*-glucan exposure is controlled by the fungal CEK1-mediated mitogen-activated protein kinase pathway that modulates immune responses triggered through dectin-1. *Infection and Immunity*.

[164] Reid DM, Gow NA, Brown GD (2009). Pattern recognition: recent insights from Dectin-1. *Current Opinion in Immunology*.

[165] Nett J, Lincoln L, Marchillo K, Andes D (2007). *β*-1,3 glucan as a test for central venous catheter biofilm infection. *Journal of Infectious Diseases*.

[166] Jesaitis AJ, Franklin MJ, Berglund D (2003). Compromised host defense on *Pseudomonas aeruginosa* biofilms: characterization of neutrophil and biofilm interactions. *Journal of Immunology*.

[167] Luo G, Ibrahim AS, Spellberg B, Nobile CJ, Mitchell AP, Fu Y (2010). *Candida albicans* Hyr1p confers resistance to neutrophil killing and is a potential vaccine target. *Journal of Infectious Diseases*.

[168] van de Veerdonk FL, Netea MG, Joosten LA, van der Meer JWM, Kullberg BJ (2010). Novel strategies for the preventionand treatment of *Candida* infections: the potential of immunotherapy. *FEMS Microbiology Reviews*.

[169] Bujdáková H, Paulovičová E, Paulovičová L, Šimová Z (2010). Participation of the *Candida albicans* surface antigen in adhesion, the first phase of biofilm development. *FEMS Immunology and Medical Microbiology*.

[170] Fujibayashi T, Nakamura M, Tominaga A (2009). Effects of IgY against *Candida albicans* and *Candida* spp. adherence and biofilm formation. *Japanese Journal of Infectious Diseases*.

[171] Dürig A, Kouskoumvekaki I, Vejborg RM, Klemm P (2010). Chemoinformatics-assisted development of new anti-biofilm compounds. *Applied Microbiology and Biotechnology*.

